# Protein–Protein Interactions Modulate the Docking-Dependent E3-Ubiquitin Ligase Activity of Carboxy-Terminus of Hsc70-Interacting Protein (CHIP)*[Fn FN2]

**DOI:** 10.1074/mcp.M115.051169

**Published:** 2015-09-01

**Authors:** Vikram Narayan, Vivien Landré, Jia Ning, Lenka Hernychova, Petr Muller, Chandra Verma, Malcolm D. Walkinshaw, Elizabeth A. Blackburn, Kathryn L. Ball

**Affiliations:** From the ‡IGMM, University of Edinburgh Cancer Research Centre, Cell Signalling Unit, Crewe Road South, Edinburgh EH4 2XR, UK;; §CTCB, Institute of Structural and Molecular Biology, University of Edinburgh, The King's Buildings, Mayfield Road, Edinburgh EH9 3JR, UK;; ¶Regional Centre for Applied Molecular Oncology, Masaryk Memorial Cancer Institute, 656 53 Brno, Czech Republic;; ‖Bioinformatics Institute (A*STAR), 30 Biopolis Street, 07-01 Matrix, Singapore 138671; Department of Biological Sciences, National University of Singapore, 14 Science Drive 4, Singapore 117543; School of Biological Sciences, Nanyang Technological University, 60 Nayang Drive, Singapore 637551

## Abstract

CHIP is a tetratricopeptide repeat (TPR) domain protein that functions as an E3-ubiquitin ligase. As well as linking the molecular chaperones to the ubiquitin proteasome system, CHIP also has a docking-dependent mode where it ubiquitinates native substrates, thereby regulating their steady state levels and/or function. Here we explore the effect of Hsp70 on the docking-dependent E3-ligase activity of CHIP. The TPR-domain is revealed as a binding site for allosteric modulators involved in determining CHIP's dynamic conformation and activity. Biochemical, biophysical and modeling evidence demonstrate that Hsp70-binding to the TPR, or Hsp70-mimetic mutations, regulate CHIP-mediated ubiquitination of p53 and IRF-1 through effects on U-box activity and substrate binding. HDX-MS was used to establish that conformational-inhibition-signals extended from the TPR-domain to the U-box. This underscores inter-domain allosteric regulation of CHIP by the core molecular chaperones. Defining the chaperone-associated TPR-domain of CHIP as a manager of inter-domain communication highlights the potential for scaffolding modules to regulate, as well as assemble, complexes that are fundamental to protein homeostatic control.

Tetratricopeptide repeats (TPR)^1^ are versatile structural modules conserved from *E. coli* to man, which function in fundamental processes such as transcriptional control, kinase signaling, protein folding and immunity (1–3). TPR-domains are composed of two antiparallel α-helices (containing a total of 34 amino acids) packed in tandem arrays to create a characteristic fold and binding cleft. Cleft formation facilitates protein–protein interactions and underpins the role of TPR-domains as molecular scaffolds for the assembly of multi-protein complexes (4, 5). Although crystallographic studies originally led to the conclusion that TPR-domains were relatively rigid structures with an invariant conformation on ligand binding, more recent studies on bacterial Rap proteins suggests that TPR-domain binding can induce gross conformational changes in the protein as a whole (6). Using nuclear magnetic resonance (NMR; (7)), circular dichroism (CD (8)) and hydrogen deuterium exchange mass spectrometry (HDX-MS (9)), the flexible character of the apo-TPR has been uncovered. This has pointed to an essential role for unstructured or intrinsically disordered TPR-domain regions in a coupled fold-on-binding mechanism. These data suggest that flexible TPR-domain structures may be an advantage when it comes to setting up protein interaction networks (10).

A subset of the TPR-domain proteins is known to associate with the Hsp70/Hsp90 family of molecular chaperones through interaction with a conserved C-terminal (EEVD) motif and act as cochaperones. CHIP (Carboxy-terminus of Hsc70-interacting protein) is an E3-ligase with three TPRs within its N terminus, a central charged domain and a C-terminal U-box that is required for E2-conjugating enzyme binding and E3-ligase activity. CHIP functions as an Hsp70 cochaperone (11), linking the molecular chaperones to the ubiquitin proteasome system. In this case, Hsp70 is proposed as a targeting subunit that also acts as a bridge between CHIP and unfolded substrates. In an alternate noncanonical pathway that has come to light over the past few years, CHIP can also interact directly with native substrates to facilitate ubiquitination. This docking-dependent substrate ubiquitination activity can impact on the steady state levels (12), localization (13) or activity (14) of the target protein.

Here, a dynamic role for the TPR-domain in the regulation of CHIP structure and function is proposed. Using physiologically relevant folded substrates such as p53 and IRF-1, we have defined the TPR-domain of CHIP as a modulator site for allosteric effectors of its U-box function and E3-ligase activity (12, 15). We discuss inherent flexibility of the TPR-domain and how this can mediate allosteric regulation of E3-ligase activity in response to chaperone interactions.

## EXPERIMENTAL PROCEDURES

### 

#### 

##### Chemicals, Antibodies, and Peptides

Antibodies were used at 1 μg/ml and were anti-IRF-1 mAb (BD Biosciences, Franklin Lakes, NJ), anti-p53 DO- 1, anti-Mdm2 4B2 and anti-CHIP v3.1 mAbs (Moravian Biotechnology, Brno, Czech Republic), anti-CHIP N-terminal pAb (Sigma, St. Louis, MO), anti-Hsp70 pAb (Stressgen, Farmingdale, NY) and anti-His mAb (Novagen, Billerica, MA). Secondary antibodies were purchased from Dako Cytomation, Carpinteria, CA. MG-132 (Calbiochem, Billerica, MA) was dissolved in dimethyl sulfoxide (DMSO) to 10 mm and used as indicated. Peptides were from Chiron Mimotopes, Melbourne, Australia and were synthesized with a Biotin-tag and an SGSG spacer at the N terminus; peptides were solubilized in DMSO. ATP was purchased from Calbiochem and creatine phosphate from Sigma.

##### Plasmids and Purified Proteins

pDEST-15-codon optimized IRF-1 (GST-IRF-1) and pET15b-CHIP (His-CHIP; wt, K30A and ΔTPR) were purified using glutathione-Sepharose (Amersham Biosciences GE, Little Chalfont, United Kingdom) and Ni^2+^-NTA agarose (Qiagen, Venlo, Netherlands) respectively, according to the manufacturer's instructions. An NdeI-codon optimized IRF-1-EcoRI fragment was amplified from pDEST-15-IRF-1, ligated into pCOLDI (TaKaRa Bio, Kusatsu, Japan) to give pCOLDI-IRF-1 (His-IRF-1) and purified as above following expression at 15 °C for 15 min by addition of IPTG (1 mm). pET3a-CHIP (untagged CHIP; wt and K30A mutant) was sub-cloned from pET15b-CHIP using NdeI and BamHI. Recombinant untagged p53 was purified as previously described (16). Purified recombinant Hsp70 was purchased from Stressgen, ubiquitin and UBE1 from Boston Biochem, Cambridge, MA and creatine phosphokinase from Sigma. Purified His-UbcH5a and His-tag cleaved UbcH5a were produced in-house. pcDNA3-IRF-1, pcDNA3-CHIP and His-Ub are as previously described (12). Purification of untagged CHIP is described in detail in the supplemental text.

##### Cell Culture

H1299 cells were cultured in RPMI 1640 (Roswell Park Memorial Institute 1640; Invitrogen, Waltham, MA) supplemented with 10% (v/v) fetal bovine serum (Autogen Bioclear, Calne, United Kingdom) and 1% (v/v) penicillin-streptomycin mix (Invitrogen), and were maintained at 37 °C/5% CO_2_. Cells were seeded 24 h before transfection and DNA transfected into the cells using Attractene (Qiagen) according to the manufacturer's recommendations.

##### HDX-MS

Deuteration of the CHIP proteins, either wt or mutant, was initiated by a sequential dilution into deuterated water with 1% DMSO to a final concentration of 1 μm. The exchange was carried out at room temperature and was quenched by the addition of 1 m HCl in 1 m glycine at 10 s, 30 s, 1 min, 5 min, 15 min, 30 min, 45 min, 1 h, and 2 h followed by rapid freezing in liquid nitrogen. Each sample was thawed and injected onto an immobilized pepsin column (15 μl bed volume, flow rate 20 μl/min, 2% acetonitrile/0.05% trifluoroacetic acid). Peptides were trapped and desalted on-line on a peptide microtrap (Michrom Bioresources, Auburn, CA) for 2 min at flow rate 20 μl/min. Next, the peptides were eluted onto an analytical column (Jupiter C18, 1.0 × 50 mm, 5 μm, 300Å, Phenomenex, CA) and separated using a linear gradient elution of 10% B in 2 min, followed by 31 min isocratic elution at 40% B. Solvents were: A, 0.1% formic acid in water; B, 80% acetonitrile/0.08% formic acid. The immobilized pepsin column, trap cartridge and the analytical column were kept at 1 °C. Mass spectrometric analysis was carried out using an Orbitrap Elite mass spectrometer (Thermo Fisher Scientific) with ESI ionization on-line connected with a robotic system based on the HTS-XT platform (CTC Analytics, Zwingen, Switzerland). The instrument was operated in the positive ion mode, and a data-dependent method was employed for peptide mapping (HPLC-MS/MS). Each MS scan was followed by MS/MS scans of the top three most intensive ions from both CID and HCD fragmentation spectra. Tandem mass spectra were searched using SequestHT search engine against the cRap protein database (ftp://ftp.thegpm.org/fasta/cRAP) containing sequence of the CHIP protein with the following search settings: mass tolerance for precursor ions of 10 ppm, mass tolerance for fragment ions of 0.6 Da, no-enzyme specificity and no-fixed or variable modifications were applied. The false discovery rate at peptide identification level was set to 1%. Sequence coverage was analyzed with Proteome Discoverer software version 1.4 (Thermo Fisher Scientific; see supplemental Table S1). Analysis of deuterated samples was done in HPLC-MS mode with ion detection in the orbital ion trap and the data were processed using HDX Workbench (17). Graphs showing deuteration kinetics were plotted using Draw-HDX-Plot (MSTools).

##### Binding Assays

Purified protein (CHIP or IRF-1, 100 ng) was immobilized on microtitre plates in 0.1 m NaHCO_3_ (pH 8.6) overnight at 4 °C. Alternately, biotin-labeled Hsp70 peptide at saturating amounts (∼60 pmol) was captured onto a microtitre plate coated with streptavidin (1 μg/well in PBS). Following washing in PBS supplemented with 0.1% (v/v) Tween-20, nonreactive sites were blocked using 3% (w/v) BSA in PBS. A titration of the protein and/or peptide of interest was added in 1× ELISA Buffer (25 mm HEPES, pH 7.5, 50 mm KCl, 10 mm MgCl_2_, 5% (v/v) glycerol, 0.1% (v/v) Tween-20) for 1 h at room temperature. Binding was detected using the stated antibodies, plus either HRP-tagged anti-mouse or HRP-tagged anti-rabbit 2°, and electrochemical luminescence was quantified using a luminometer.

##### AlphaScreen

*A*mplified *L*uminescent *P*roximity *H*omogeneous *A*ssays (AlphaScreen) were carried out in white half-area microtitre plates according to the manufacturer's recommendations. In brief, biotin-tagged Hsp70 peptide (GPTIEEVD; 6.25 ng) was linked to streptavidin donor beads (20 μl) diluted 1:100 and incubated with a titration (0–100 ng in 10 μl volume) of His-CHIP wt or K30A mutant conjugated to protein-A acceptor beads (20 μl of 1:100 dilution) using anti-His mAb. The reaction mix was incubated for 1 h at room temperature and quantified using an EnVision fluorescence detector (Perkin Elmer, Waltham, MA). For His-UbcH5:untagged CHIP AlphaScreen, the assay was performed as above except that His-tagged UbcH5a (50 ng) was anchored onto Nickel-chelate donor beads and a titration (0–100 ng) of untagged CHIP wt or K30A onto protein-A acceptor beads using anti-CHIP N-terminal pAb.

##### Ubiquitination Assays

Cell-based ubiquitination assays were carried out as previously described (18). *In vitro* ubiquitination assays (19) were started with His-CHIP (50–100 nm) or untagged CHIP (100–200 nm), incubated for up to 20 min as indicated at 30 °C, and stopped by the addition of SDS-PAGE sample buffer. Samples were analyzed using 4–12% NuPAGE gels in a MOPS buffer system/immunoblot. If required, Hsp70 (1:1 molar ratio with CHIP unless stated otherwise) and/or Hsp40 (at 1:10 ratio of Hsp40:Hsp70) or peptides were added to the ubiquitination mix (see figure legends for details) immediately prior to the incubation at 30 °C.

##### E2-Discharge Assay

Reactions contained 25 mm HEPES pH 8.0, 10 mm MgCl_2_, 350 nm ATP, 0.5 mm DTT, 0.05% (v/v) Triton X-100, 0.25 mm benzamidine, 10 μm ubiquitin, 100 nm UBE1, and 1 μm UbcH5a (E2). The E2 was charged for 15 min at 30 °C after which His-CHIP (0–200 nm; ±Hsp70 peptide as required) was added and reactions were incubated for a further 15 min at 30 °C to discharge the E2. To stop the reaction, SDS-PAGE sample buffer (without DTT, but with 2.5 mm N-ethylmaleimide) was added and the reactions analyzed on 4–12% NuPAGE gels/immunoblot.

##### Thermal Unfolding and Differential Scanning Calorimetry

SYPRO Orange was diluted to 50× in Buffer S (20 mm Tris, pH 8, 150 mm NaCl) and used at 5X. His-CHIP wt or K30A was diluted to 5 μm in Buffer S before the addition of SYPRO Orange. Hsp70 peptides (or a DMSO control) were added to a final concentration of 5 μm. Samples were loaded on a 96-well PCR plate (50 μl per reaction) and sealed. Unfolding was measured using an iCycler iQ Real-Time PCR system (Bio-Rad, Hercules, CA) by heating samples from 25 °C to 60 °C at 1 °C increments with a 30 s incubation at each increment. Fluorescence intensity was measured in relative fluorescent units (RFU) using excitation/emission wavelengths of 485 nm/575 nm. All samples were repeated in triplicate.

Differential scanning calorimetry experiments were performed using a MicroCal VP-capillary DSC system (GE Healthcare). Proteins were exchanged into degassed 50 mm HEPES, pH7.5, 150 mm sodium chloride, 1 mm DTT to a final concentration of 6.5 μm prior to analysis. CHIP was heated from 5 to 85 °C at a scan rate of 60 °C/hour. Buffer was scanned under the same conditions to provide a buffer baseline. All samples were repeated in triplicate. Data were normalized for concentration and baseline corrected. Thermograms were analyzed with the software provided by the manufacturer.

##### Limited Proteolysis

CHIP protein (2 μg; plus 4 μg peptide if required) was incubated with Glu-C (Roche; 40 ng) in 25 mm ammonium carbonate (pH 7.8) at room temperature as indicated. Reactions were stopped by addition of sample buffer and heating at 85 °C for 5 min. Samples were analyzed by 4–12% NuPAGE gels and stained with InstantBlue (Expedeon, San Diego, CA). For tryptic digests, 500 ng CHIP proteins, 5 ng trypsin (Roche, Basel, Switzerland), and 1 μg peptide was used, and the incubation carried out in 100 mm Tris-HCl (pH 8.5) at 4 °C.

##### MD Simulations

The crystal structure of mouse CHIP in complex with Hsp90 peptide (PDB code 2C2L, resolved at 3.3 Å (20)) was used as the initial structure for simulations. Five mutations (P77H, T167S, H188D, G192S, I194V - mouse numbering) were introduced into the crystal structure to obtain human CHIP using the WHATIF (http://swift.cmbi.ru.nl/whatif/) program. Simulations were then run on three systems: the CHIP dimer with Hsp90 pep (chains A,B,E,F from 2C2L where chains A and B are the CHIP dimer and chains E and F are the peptides bound to chains A and B respectively), the CHIP dimer without peptide (chains A,B) and the CHIP dimer with Lys^30^ mutated to Ala (chains A,B). Further information is given in the supplementary methods.

## RESULTS

### 

#### 

##### Hsp70 Modulates the E3-Ligase Activity of CHIP

**Fig. 1.**
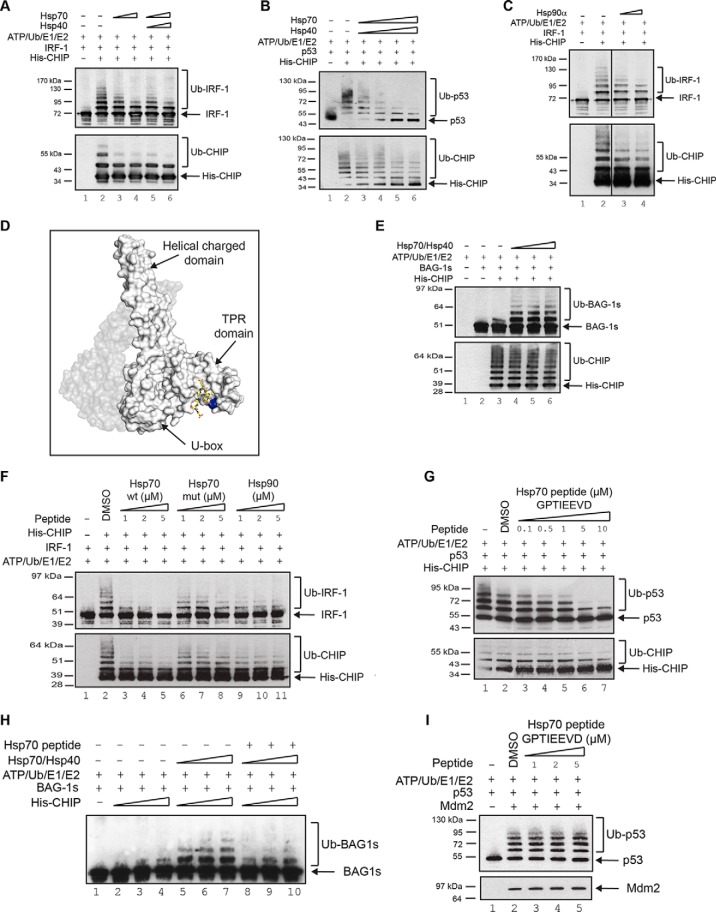
**Hsp70 differentially modulates CHIP-dependent ubiquitination.** (**A, C**) Immunoblot of *in vitro* ubiquitination reactions assembled using ATP, ubiquitin, UBE1, UbcH5a, His-CHIP and GST-IRF-1 in the presence of a titration of Hsp70 with or without Hsp40 (**A**) or Hsp90 (**C**) at either a 1:1 or 1:2 molar ratio of Hsp70/Hsp90 with CHIP. (**B, E**) Immunoblot of *in vitro* ubiquitination assays assembled as in (**A**) except using untagged p53 (**B**) or GST-BAG-1s (**E**) as substrate, in the presence of Hsp70 and Hsp40. (**D**) Snapshot of the crystal structure of mCHIP dimer (protomers in shades of gray) in complex with Hsp90 peptide (yellow sticks; PDB code 2C2L) generated using PyMOL v1.4.1. Lys^30^ is highlighted in blue. (**F, G**) Immunoblot of *in vitro* ubiquitination reactions assembled using ATP, ubiquitin, UBE1, UbcH5a, His-CHIP and His-IRF-1 (**F**) or untagged p53 (**G**) in the presence of a titration of Hsp70 (wt: GPTIEEVD; mut: GAAAEEVD) or Hsp90 (DTSRMEEVD) peptide as indicated. A carrier only control (DMSO) was included. (**H**) As above, except that GST-BAG-1s was used as the substrate and both full-length Hsp70/Hsp40 as well as Hsp70 wt peptide were included in the assay as indicated. (**I**) As in (**G**) except using GST-Mdm2 as the E3 ligase.

We are interested in the emerging noncanonical activity of CHIP where the E3-ligase interacts with, and ubiquitinates, native-folded proteins, thereby regulating their steady state levels, localization and/or activity during normal growth control and cellular stress (12–14). In the canonical pathway, where CHIP acts as a link between the molecular chaperones and the ubiquitin proteasome system, the TPR-domain binds to a well-defined consensus motif in the C terminus of Hsp70 or Hsp90, facilitating the ubiquitination of client proteins (13). However, whether the core molecular chaperones modulate the activity of CHIP as a docking-dependent ligase (12) for native proteins has not been addressed. Initial experiments therefore concentrated on determining the E3-ligase activity of CHIP in the apo-form compared with CHIP in an Hsp70-bound conformation using docking-dependent substrates. When the effect of Hsp70 on CHIP-mediated ubiquitination was determined using p53 and IRF-1 as substrates (12, 21), we found that pre-incubation of CHIP with Hsp70, alone or together with its physiological partner Hsp40, inhibited substrate ubiquitination (Fig 1*A* and 1*B*). Furthermore, addition of Hsp90 to the ubiquitination assay also inhibited IRF-1 modification by CHIP (Fig 1*C*), suggesting that the conserved TPR-interacting motif (EEVD-motif; Fig 1*D*) at the C terminus of both Hsp70 and Hsp90 might be involved in CHIP regulation.

Consistent with previous observations (22), under conditions where Hsp70 inhibited CHIP-dependent ubiquitination of IRF-1 and p53, it stimulated the modification of a well-defined Hsp70 cochaperone BAG-1s (Fig 1*E*). Further, although Hsp70 inhibited CHIP auto-ubiquitination in the presence of either IRF-1 or p53 (Fig 1*A* and 1*B*; lower panels), no inhibition of CHIP auto-ubiquitination was detected in the BAG-1s assay (Fig 1*E*; lower panel). Thus, Hsp70 can act as an activator or inhibitor of CHIP E3-ligase activity dependent on the substrate.

The above data suggest that Hsp70 can inhibit ubiquitination of p53 and IRF-1 through its interaction with the TPR-domain of CHIP. However, in addition to its ability to bind CHIP, Hsp70 can also bind directly to p53 and IRF-1 (23, 24). Although a recent study on the isolated TPR-domain of CHIP suggests that both the C terminus of Hsp70 and a region from the Hsp70 lid (25) contact CHIP, studies on full-length CHIP suggest that it interacts exclusively with the conserved C terminus of Hsp70 (9). Thus, to extend our analysis, we used a C-terminal peptide from Hsp70 (^634^GPTIEEVD^641^ or ^633^SGPTIEEVD^641^) that binds exclusively to the TPR-domain of CHIP (Fig 1*D* and supplemental Fig. S1*A*, S1*B*) and not to its substrates. When CHIP was preincubated with the Hsp70 peptide, ubiquitination of IRF-1 was reduced compared with a mutant peptide control (GAAAEEVD; Fig 1*F* and supplemental Fig. S1*C*). Similarly, a peptide based on the C terminus of Hsp90 (^724^DTSRMEEVD^732^) also inhibited ubiquitination of IRF-1. Consistent with data showing the Hsp90 peptide binds more weakly to CHIP than the Hsp70 peptide (supplemental Fig. S1*D*), it was less effective as an inhibitor of IRF-1 ubiquitination (Fig 1*F*, lanes 9–11). Ubiquitination of p53 (Fig 1G) and CHIP auto-ubiquitination (Fig 1*F* and Fig 1*G* lower panels) were also inhibited by the Hsp70-peptide. Of interest was data showing that Hsp70/40 stimulated ubiquitination of BAG-1s was still suppressed by the Hsp70 peptide (Fig 1H), suggesting that the architecture of the CHIP:BAG-1s:Hsp70 complex may be different to that of the complexes containing docking dependent substrates. As a control for peptide specificity in binding to CHIP, we show that it has no effect on the activity of the MDM2 E3-ligase in a p53 ubiquitination assay (Fig 1*H*) where all the components of the assay (with the exception of the E3) were otherwise identical to those in Fig 1*G*.

##### CHIP-K30A has an Intrinsic Defect in E3-Ubiquitin Ligase Activity

The data presented above demonstrate that binding of an Hsp70-based peptide ligand to the TPR-domain of CHIP was sufficient to modulate its docking-dependent E3-ligase activity. Recent solution studies show that, in contrast to the highly flexible nature of the apo-CHIP TPR-domain, the Hsp70-bound or Hsp70 peptide-bound form of the TPR is structured and has reduced flexibility (9). We therefore hypothesized that the transition from a highly flexible to a more structured TPR form could affect the catalytic activity of CHIP.

**Fig. 2.**
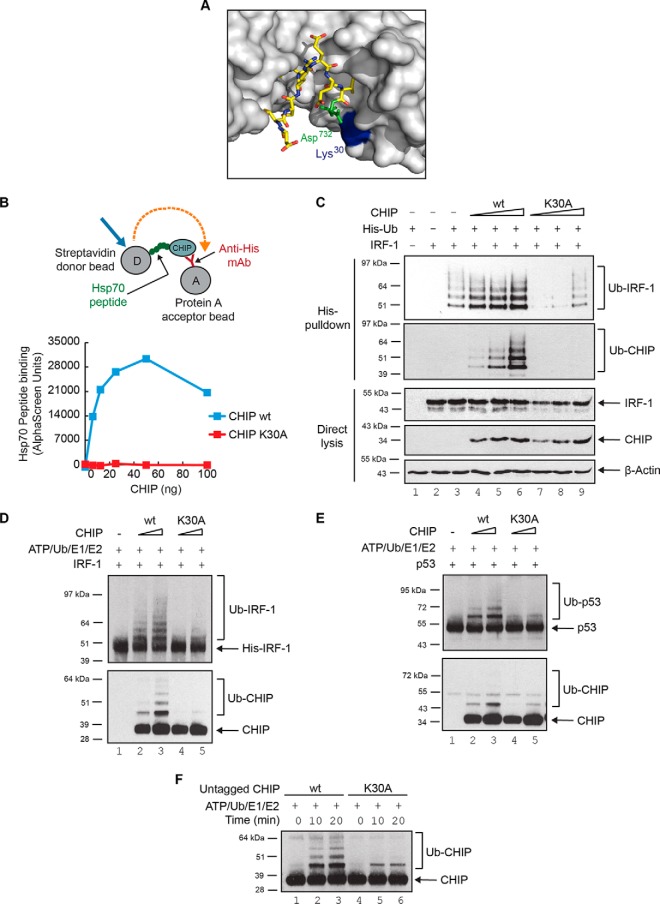
**CHIP-K30A is intrinsically defective in E3-ligase activity.** (**A**) Close-up of the Hsp90 binding site on CHIP extracted from the crystal structure of mCHIP dimer (protomers in shades of gray; also see Fig 1D) in complex with Hsp90 peptide (yellow sticks; PDB code 2C2L) generated using PyMOL v1.4.1. Lys^30^ on CHIP and Asp^732^ on Hsp90 are highlighted in blue and green respectively. (**B**) An AlphaScreen assay was set up (see cartoon) to measure binding dynamics of His-CHIP wt or K30A mutant with biotin-tagged Hsp70 peptide (GPTIEEVD) in solution. (**C**) Ubiquitination of exogenous IRF-1 in H1299 cells transiently transfected with plasmids encoding CHIP wt or K30A mutant and His-tagged ubiquitin. Immunoblots show ubiquitinated protein (His-pulldown) and total protein (Direct lysis). (**D, E**) *In vitro* ubiquitination assays were assembled using ATP, ubiquitin, UBE1, UbcH5a, untagged CHIP wt or K30A, and His-IRF-1 (**D**) or untagged p53 (**E**) as substrate. Reactions were analyzed by 4–12% NuPAGE/immunoblot. (**F**) Immunoblot of *in vitro* ubiquitination assays assembled as above except in the absence of substrate to study auto-ubiquitination of untagged CHIP wt or K30A proteins over time.

We reasoned that mutation of certain TPR-domain residues to Ala, a residue that encourages helix formation (26), may mimic the stabilizing effect of Hsp70-binding on the TPR-domain. Lys^30^ of CHIP is one of two basic residues (the other being Lys^95^) that are required to form a dicarboxylate clamp around the C-terminal Asp of Hsp70/90 (Fig 2*A*), and mutation of this residue to Ala has been predicted to prevent Hsp70 binding. The Lys^30^→Ala (K30A) point mutant protein may therefore provide a tool to study the effect of stabilizing the TPR-domain in the absence of added ligand.

Following expression, purification (supplemental Fig. S2*A*) and normalization of K30A and wild-type CHIP (supplemental Fig. S2*A*, S2*B*), we verified that the K30A mutant was folded and predominantly dimeric using biophysical techniques including dynamic light scatter (supplemental Fig. S2*C*) and size exclusion chromatography (supplemental Fig. S2*D*). We then asked whether the K30A mutation produced protein that was deficient in binding to Hsp70. CHIP-K30A protein was unable to bind to a C-terminal peptide from Hsp70 (Fig 2*B*) in a real-time AlphaScreen assay under conditions where the wild-type protein bound with a high affinity. As CHIP-K30A constructs have been used extensively in cell-based assays to study the chaperone-dependence of CHIP (27–29), we next determined the effect of the Lys^30^ substitution on IRF-1 modification in cells. In-cell ubiquitination assays showed that the over-expression of wild-type CHIP markedly enhanced IRF-1 modification by ubiquitin (Fig 2*C*, compare lanes 6 and 3) whereas CHIP-K30A did not; rather, the mutant had some dominant-negative activity toward endogenous E3-ligases. This result could be interpreted as a requirement for Hsp70 in enhanced substrate ubiquitination. However, we also noted that CHIP-K30A did not undergo auto-ubiquitination (Fig 2*C*; Ub-CHIP), suggestive of differences in its intrinsic activity in a way which, as predicted, might reflect a stabilization of the TPR-domain structure by Ala.

To determine if CHIP's intrinsic E3-ligase activity was affected by the TPR-domain mutation (K30A), the mutant protein was assayed alongside the wild-type. To rule out an effect of the N-terminal His-tag on the structure and activity of the TPR, these experiments were carried out using untagged CHIP (supplemental Fig. S2*A*). Strikingly, CHIP-K30A displayed a significant reduction in its E3-ligase activity compared with the wild-type protein using either IRF-1 (Fig 2*D*) or p53 (Fig 2*E*) as the substrate. In addition, in keeping with the cell-based assays (Fig 2*C*), the CHIP-K30A mutant was severely restricted in its ability to undergo auto-ubiquitination (Fig 2*F*). As the *in vitro* ubiquitination assay does not contain Hsp70/90, the decrease in CHIP-K30A E3-activity is not because of loss of Hsp70-binding potential.

##### Evidence of TPR-Mediated Changes in CHIP Conformation

Data presented above suggest that the TPR-domain potentially plays an active role in the regulation of CHIP's E3-ligase activity, and that modulation by ligand binding or the introduction of structure stabilizing amino acids may result in a shift in the protein ensemble that impacts the activity of the U-box. In addition, as Hsp70 and Hsp70 peptide, or the introduction of a Lys^30^ point mutation within the TPR, have similar effects on the activity of CHIP, we hypothesized that the CHIP-K30A mutation might 'mimic' binding of Hsp70/90 to the ligase. To test our hypothesis, we investigated whether CHIP-K30A had different dynamic properties and if these were similar to those of Hsp70-bound CHIP.

**Fig. 3.**
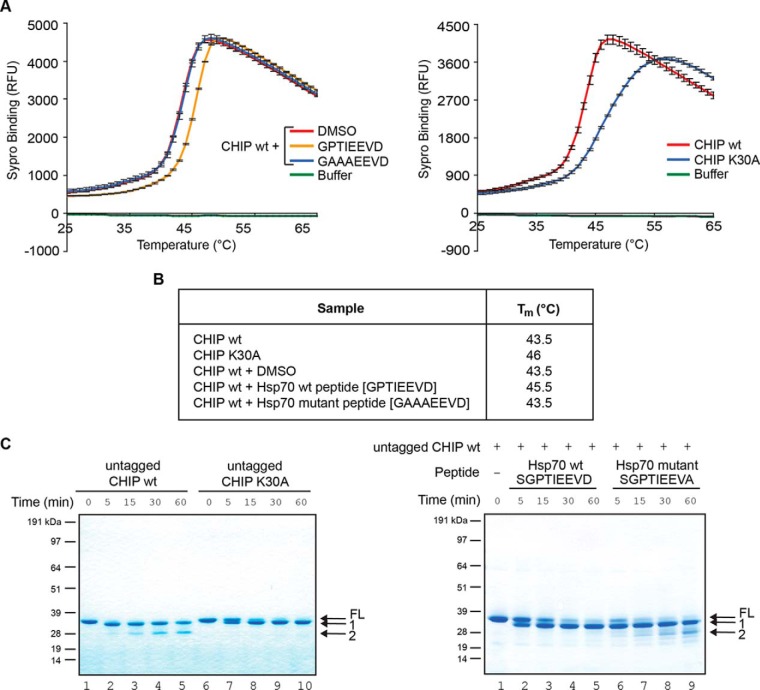
**CHIP-K30A and Hsp70-bound CHIP are conformationally distinct from the wild-type protein.** (**A**) Graph showing the unfolding of His-CHIP wt pre-incubated with the indicated peptides based on Hsp70 (left panel) or His-CHIP wt or K30A mutant (right panel) as a function of temperature change measured by the uptake of the fluorescent dye SYPRO Orange. Shown is the means ± S.E. of mean of 3 experiments. (**B**) Table listing the mid-point temperature of phase transition (T_m_) of each sample in (**A**) that was calculated by plotting the gradient of protein unfolding against the temperature gradient [-d(RFU)/dT]. (**C**) InstantBlue stained gel of untagged CHIP wt or K30A (left panel) digested with the protease Glu-C. FL is the full-length protein and band 1 is a cleavage product that persists in the K30A mutant. Band 2 is only observed in digests of the wt protein. Also shown is a Glu-C digest of His-CHIP wt protein in complex with wt or mutant Hsp70 peptides (right panel).

We started by determining whether peptide binding and/or Lys^30^ substitution affected the melting temperature (T_m_) of full-length CHIP using fluorescence-based thermal shift assays as a measure of TPR secondary-structure and folding. CHIP had a higher melting temperature when bound to the Hsp70 peptide than in the un-liganded state (Fig 3*A* left panel and Fig 3*B*; T_m_ unbound (DMSO) = 43.5 °C, and bound [wt peptide; GPTIEEVD] = 45.5 °C) or in the presence of the low affinity mutant peptide (Hsp70 mutant peptide; GAAAEEVD, supplemental Fig. S1*A*, S1*B*). Strikingly, when wild-type CHIP was compared with CHIP-K30A (Fig 3*A* right panel and Fig 3*B*), the mutation, like ligand binding, made the protein more resistant to melting, with a T_m_ for CHIP-K30A of 46 °C. The data support the concept that substituting Lys^30^ with Ala encourages a more structured or folded conformation to be adopted by the CHIP TPR-domain.

Next, limited proteolysis was used to probe for differences in the conformation of liganded- and apo-CHIP compared with the CHIP-K30A mutant protein. Conditions from preliminary experiments using Glu-C or trypsin (supplemental Fig. S3) were used to compare wt- and CHIP-K30A proteins digested with Glu-C to CHIP in the presence of the active Hsp70 wt or mutant peptides (Fig 3*C*). In this case, the mutant control peptide used had the sequence SGPTIEEVA and was chosen as it binds CHIP only weakly and as a result is not able to inhibit CHIP E3-activity (supplemental Fig. S1*C*). The results showed a striking similarity between the banding pattern seen over-time for the CHIP-K30A mutant and for liganded CHIP *i.e.* full-length protein was more resistant to cleavage and no band 2 was generated (Fig 3*C*). On the other hand, the bands generated for the wt protein in the absence of ligand or in the presence of control peptide were similar, with band 2 appearing between 5 and 15 min. Together the data suggest that the CHIP-K30A and Hsp70 peptide-bound forms of CHIP have less structural flexibility and are in a more 'ordered' or compact form than wild-type CHIP when in solution.

##### Striking Similarity between the Structures of Liganded and Mutant CHIP

**Fig. 4.**
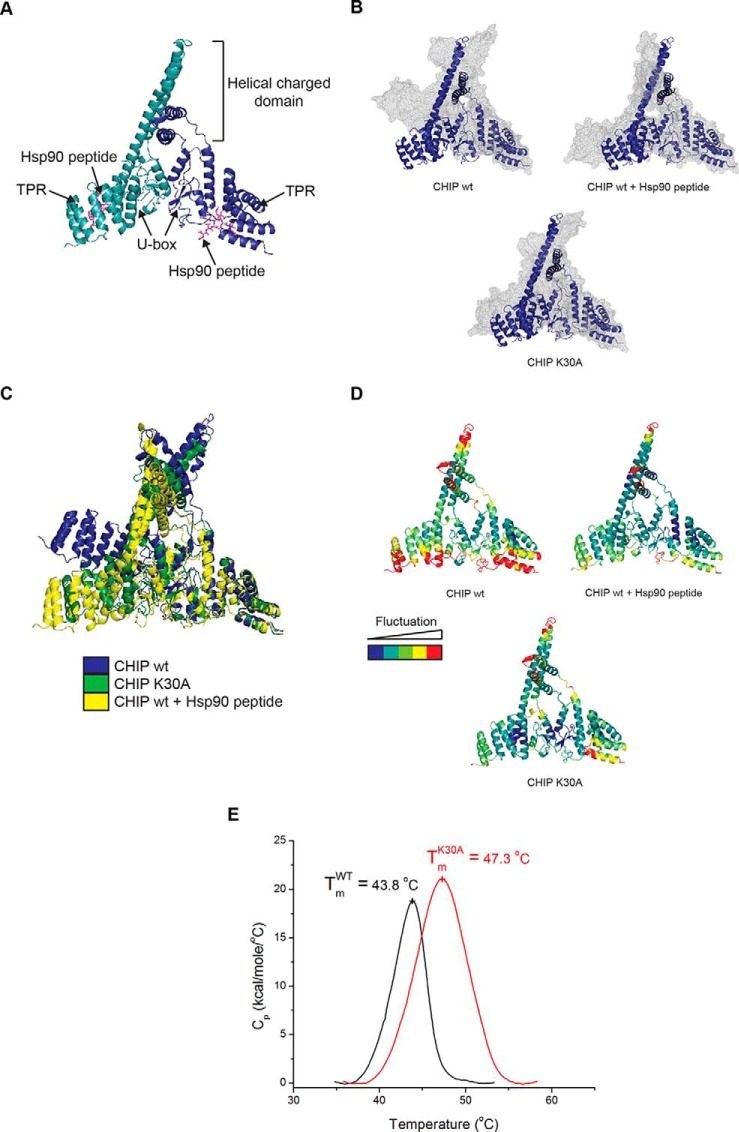
**CHIP-K30A and Hsp70-bound CHIP have similar equilibrium structures.** (**A-D**) Images were generated using PyMOL v.1.4.1. (**A**) Crystal structure of murine CHIP dimer (monomers in shades of blue) in complex with Hsp90 peptide (pink sticks; adapted from PDB 2C2L). (**B**) Overlay of the CHIP dimer before (blue ribbon) and after (gray mesh) 20 ns MD simulations for unliganded CHIP wt (upper left), CHIP wt in complex with Hsp90 peptide (upper right) and CHIP-K30A (bottom). (**C**) Overlaid snapshots of the CHIP dimer in apo and liganded forms and with Lys^30^ mutated to Ala after 20 ns MD simulations (from (**B**)). (**D**) Root mean square fluctuation (RMSF) of Cα obtained from the trajectories of the 20 ns simulations of CHIP wt ± peptide and the CHIP-K30A mutant. The score of the positional fluctuation analysis averaged over amino acid were color coded and indicated on the crystal structure. (**E**) For differential scanning calorimetry, protein and buffer controls were heated at a rate of 60 °C/hour from 5 to 85 °C. The thermal transition mid-point (T_m_) and specific heat capacities (C_p_) were determined using the instrument software (Origin, version 7.0).

To gain further insight into how the TPR-domain might mediate changes in the activity and structure of CHIP, molecular dynamics (MD) simulations were carried out using information derived from the crystal structure of mouse CHIP (residues 25–304) bound to the C-terminal Hsp90α peptide, DDTSRMEEVD (PDB code: 2C2L; Fig 4*A*). To relate the modeling to our experimental data, five mutations were introduced into the crystal structure to obtain human CHIP (see Methods). Simulations were run on dimeric CHIP protein with and without Hsp90 peptide and on the Lys^30^ mutant (Fig 4*B* and supplemental Fig. S4*A*, S4*B*). Simulations where the Hsp90 peptide was replaced with that from Hsp70 (supplemental Fig. S4*C*) were also run. The results of the simulations demonstrate that the conformation of CHIP in its liganded (Fig 4*B*, CHIP wt + Hsp90 peptide and supplemental Fig. S4*C*, CHIP + Hsp70 peptide) or mutant state (Fig 4*B*; CHIP-K30A), are very similar to each other and are different from the apo-state (Fig 4*B*; CHIP wt). In the apo-state, the protein adopts a more linear and extended conformation with gross outwards movement of both TPR-domains (supplemental Movie S1). In contrast, in both its mutant and peptide-bound states, the protein adopts a closed conformation that is similar to the crystal structure (Fig 4*C*).

Averaging the fluctuation of each residue in the CHIP structure showed that wild-type unliganded-CHIP (Fig 4*D*; upper left panel) was characterized by larger and more widespread movements than peptide-bound (upper right panel) or Lys^30^-mutant CHIP (lower panel). This suggests that the dynamics of the apo-state are different from the dynamics of the ligand-bound or mutant states, which in turn are similar to each other. These results are in good agreement with HDX-MS data showing that apo-CHIP protein is more flexible than the peptide-bound forms (9). Thus, MD simulations are consistent with experimental observations showing that CHIP has a lower melting temperature and is more susceptible to limited proteolysis in its unliganded form.

The striking similarity between CHIP when it is bound to Hsp70/Hsp90 peptides or when it contains an Ala substitution at Lys^30^ confirms that although CHIP-K30A has been studied as a nonchaperone binding mutant of CHIP (27–29), its biophysical properties are in fact like those of a constitutively Hsp-bound form. The side-chain of Lys^30^ does not appear to make any hydrogen bonds with other protein atoms during the MD simulations and is instead well hydrated. We speculate that, consistent with studies showing alanine residues favor the formation of ordered helical structures (26), mutation of Lys^30^ to the much smaller and more hydrophobic Ala, will make this region less hydrated and more likely to fold into an ordered structure.

To test the conclusions from the MD simulations experimentally, we carried out differential scanning calorimetry (Fig 4*E*). Wild-type and K30A mutant CHIP unfolded in a single melting transition with a T_m_ of 43.8 and 47.3 °C, respectively. This suggests that melting of the constituent domains is a cooperative process. K30A mutant CHIP has a notably larger enthalpy of unfolding when compared with the wild-type protein, indicative of K30A-CHIP having an increased number of hydrogen/van der Waals bonds. These data support the MD modeling which suggests that the mutant form of CHIP is less flexible than its wild-type counterpart.

##### The TPR-domain Affects U-box Activity and Substrate Docking

**Fig. 5.**
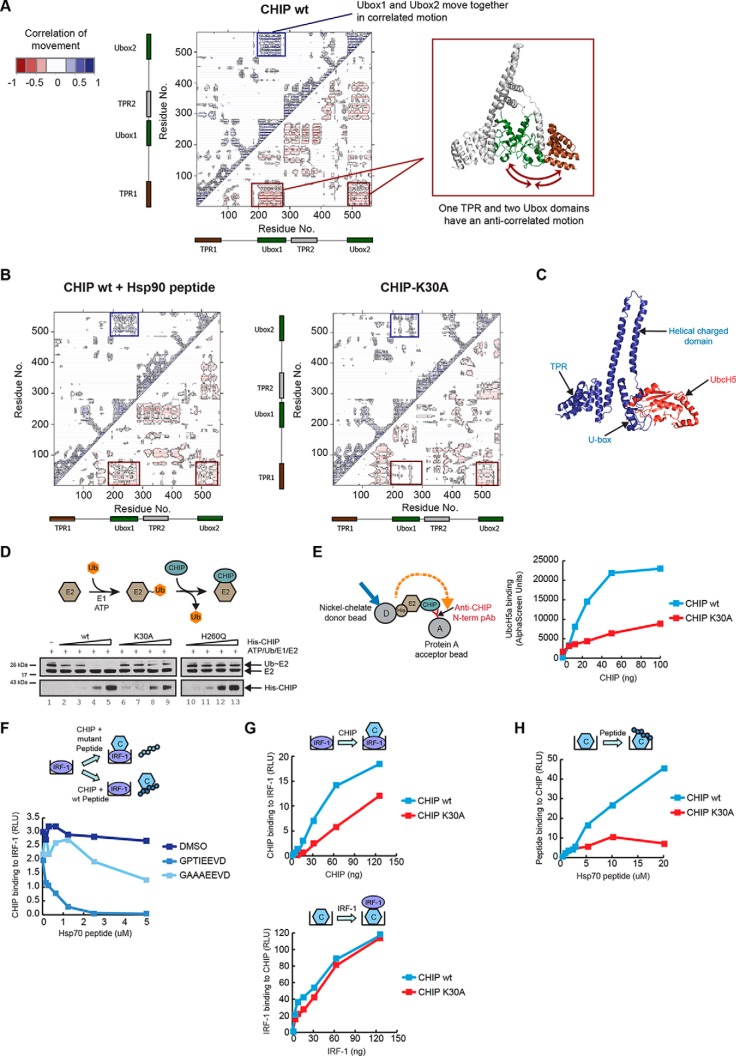
**Coordinated movements between the TPR and U-box regulate CHIP activity.** (**A**) Dynamic cross-correlation map (left panel) of Cα atoms for the un-liganded wt CHIP dimer. Correlated motions are represented above the diagonal in blue and anticorrelated below in red. Correlated movements of the CHIP U-boxes are indicated by a blue box. Anticorrelated movements of the TPR domain (right panel in brown) with both U-boxes (right panel in green) are indicated with red boxes. Cartoon of CHIP dimer (right panel) was generated using PyMOL v.1.4.1. (**B**) As above except the dynamic cross-correlation maps of Cα atoms are for Hsp90 peptide bound wt CHIP dimer (left panel) and the K30A mutant CHIP dimer (right panel). (**C**) Snapshot of the crystal structure of zebrafish CHIP-Ubox in complex with UbcH5 (from PDB 2OXQ) superimposed onto the crystal structure of mouse CHIP (from PDB 2C2L). The image, showing a single CHIP monomer, was generated using PyMOL v1.4.1. Blue ribbon: CHIP; red ribbon: UbcH5. (**D**) His-UbcH5a was charged with ubiquitin (Ub∼E2; thiolester linkage) by incubating with UBE1 and ubiquitin in the presence of ATP, following which ubiquitin discharge from the E2 by His-CHIP wt or K30A mutant was monitored. The E2-binding-defective mutant H260Q was included as a control. Shown is an immunoblot probed for CHIP and the E2. (**E**) An AlphaScreen assay was set up (see cartoon) to measure binding dynamics of untagged CHIP wt or K30A mutant anchored on protein A acceptor beads with His-tagged UbcH5a captured on Nickel-chelate donor beads in solution. (**F-G**) GST alone controls showed negligible binding and are therefore not indicated on the graphs. (**F**) Binding assay with fixed amounts of GST-IRF-1 immobilized on microtitre wells. Fixed amounts of His-CHIP wt together with a carrier control (DMSO) or a titration of Hsp70 wt or mutant peptide was added in the mobile phase. CHIP binding to IRF-1 was measured on a luminometer using an anti-CHIP antibody. (**G**) Upper panel: Binding assay as in (**F**) except that a titration of His-CHIP wt or K30A mutant was added in the mobile phase. Lower panel: Binding assay with fixed amounts of His-CHIP wt or K30A mutant coated on microtitre wells and a titration of GST-IRF-1 added in the mobile phase. (**H**) Binding assay as in (**G**) (lower panel) except that a titration of Hsp70 wt peptide was added in the mobile phase. Binding was detected using streptavidin-HRP.

When the correlations between the fluctuations for residues in all three of the CHIP simulations (Fig 4*D*) were examined, a striking anticorrelated movement (Fig 5*A*) was seen between the TPR-domain of one CHIP wild-type protomer with the U-boxes of both dimer components. This motion was strongly suppressed upon peptide binding and almost completely lost in CHIP-K30A (Fig 5*B*). Additionally, correlated motions were observed between the two U-box domains of the dimer (Fig 5*A*) in the wild-type conformation and again these were attenuated upon peptide binding or substitution of Lys^30^ (Fig 5*B*). The MD simulations therefore provide support for a model where cross-talk between distinct domains of CHIP is likely to underpin its function. Previous studies have concluded that the CHIP dimer is asymmetric and that the U-box of one of the protomers is unavailable for E2 binding because of the location of its cognate TPR-domain, whereas the U-box from the other protomer remains accessible to the E2, with only one E2-charged U-box required for CHIP E3-activity. The MD experiments suggest that changes in TPR-domain and U-box motion would not affect the ratio of E2-binding. We conclude therefore that the loss of anticorrelated motions of the two U-box domains with one of the TPR-domains (Fig 5*A* and 5*B*) upon peptide binding or Lys^30^ mutation is evidence that the TPR-domain is acting as a binding-site for allosteric effectors which negatively regulate CHIP activity. In our model, loss of anticorrelated motion would impact on the dynamic nature of the U-box rather than altering the accessibility of one, or other, of the U-boxes at any given time.

To seek experimental evidence to support the allosteric regulation of the U-box through the TPR-domain of CHIP suggested by MD, E2∼Ub-discharge assays were used. The E2-enzyme UbcH5 can act as the catalytic module for CHIP, as binding to the U-box (Fig 5*C*) generates allosteric changes in UbcH5 which facilitate substrate ubiquitination or the transfer of ubiquitin to other ubiquitin molecules (30, 31). To determine if TPR-domain-initiated changes in CHIP structure are transmitted to the U-box, we set up an E2-discharge assay and followed the loss of ubiquitin from thiolester-linked E2-ubiquitin (E2∼Ub) in response to CHIP (Fig 5*D*; Cartoon). Whereas increasing amounts of wild-type CHIP stimulated ubiquitin discharge from UbcH5 (Fig 5*D*; lanes 4 and 5) the CHIP-K30A mutant protein had a significantly reduced ability to stimulate ubiquitin loss from the E2∼Ub complex. In fact, the activity of the CHIP-K30A mutant was intermediate between that of wild-type CHIP and a U-box mutant (H260Q) that can no longer interact with the E2. When the ability of CHIP-K30A to bind UbcH5 was determined using an AlphaScreen assay (Fig 5*E*), it bound with a significantly lower affinity than wt CHIP. Thus, introduction of the structure stabilizing K to A mutation at Lys^30^ inhibits the ability of the ligase to activate UbcH5 through changes in its binding affinity.

The precise binding site(s) for IRF-1 on CHIP is not known; however, although the TPR-domain is completely dispensable for CHIP:IRF-1 complex formation, both the charged domain and U-box are required (12). Therefore, we next asked whether TPR-domain driven conformational changes in CHIP affected substrate-binding. Using protein interaction assays, we found that wt Hsp70 peptide was able to compete with IRF-1 for binding to CHIP when the ligase was in the mobile phase (Fig 5*F*). As the TPR-domain is not required for IRF-1 binding to CHIP (12), the result suggests that the peptide bound conformation of CHIP has a lower affinity for IRF-1 than CHIP in its unliganded conformation. To confirm this hypothesis, we turned to the K30A-CHIP mutant. When IRF-1 was immobilized and CHIP was in the mobile phase, K30A-CHIP binding to IRF-1 was impaired (Fig 1*G*; upper panel). However, when the assay was reversed and CHIP was immobilized on the plate, the wt and K30A-mutant proteins bound equally well to IRF-1 (Fig 1*G*; lower panel). Under the same conditions, as expected, CHIP-K30A bound poorly to Hsp70 peptide when compared with wt CHIP (Fig 1*H*). Taken together, the data suggest that (1) CHIP in its unliganded flexible form binds better to IRF-1 than in its Hsp70-bound conformation; (2) decreased binding of liganded CHIP or K30A-CHIP to IRF-1 reflects a difference in conformation rather than a direct effect of peptide binding or Lys^30^ mutation on IRF-1 binding; and (3) consistent with previous data (12), IRF-1 and Hsp70 peptide do not compete for binding to the same site on CHIP. Thus, the TPR-domain of CHIP can regulate its E3-activity through effects on both substrate and E2 binding.

##### Using HDX-MS to Define TPR-generated Changes in the U-box

**Fig. 6.**
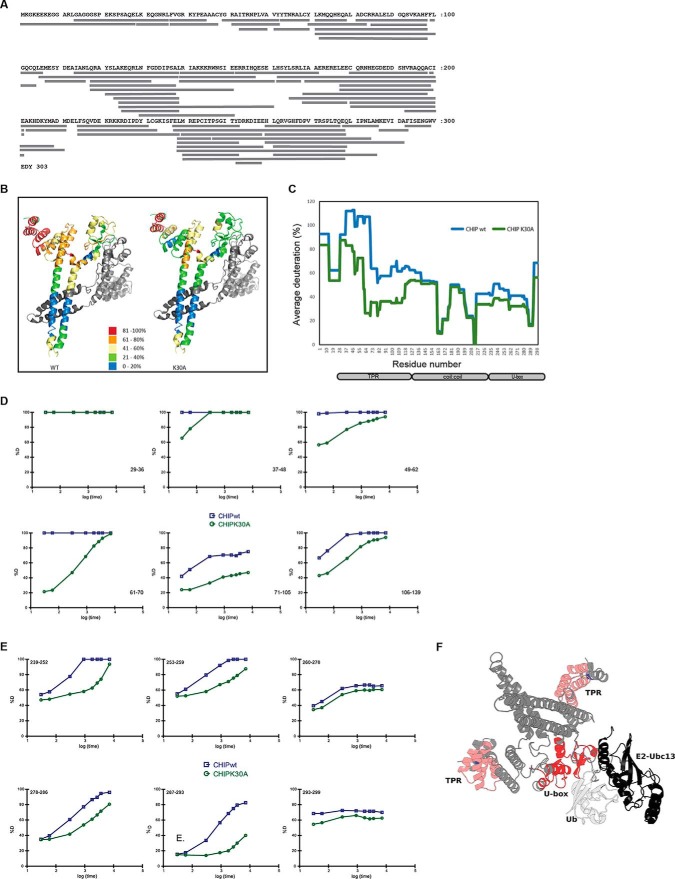
**Mapping conformational changes using HDX-MS.** (**A**) Sequence of human CHIP showing the distribution of the 67 peptides identified in the HDX-MS analysis. (**B**) The % deuteration of a given peptide from the 60s analysis was mapped onto the crystal structure of mCHIP (PDB 2C2L). Shown is the data for wt CHIP (left) and K30A CHIP (right). (**C**) Graph showing the average deuteration (%) of single amino acids of CHIP wt (blue) or K30A (green) at the 60s time point calculated as described in (32). (**D**) Graphs showing the kinetics of deuteration for selected TPR-domain peptides from wt (blue) and K30A (green) CHIP. Amino acid number is given in the bottom right hand side of the individual graphs. (**E**) As in (**D**) except that the selected peptides were from the U-box. Amino acid numbers are shown at the top left hand corner. (**F**) Structural representation of CHIP dimer in complex with Ubc13 (black) and ubiquitin (white), showing the predicted orientation of the U-box in the activated E2 complex. Shown is the structure of nearly full-length dimeric CHIP (PDB 2C2L) aligned with the CHIP U-box and Ubc13 complex (E2; PDB 2C2V), followed by alignment with ubiquitin from the complex of RING domain dimer Rnf4, E2 conjugating enzyme Ubch5a and ubiquitin (PDB 4AP4). U-box core residues (red) and N-terminal helix (salmon pink) that have reduced flexibility in the K30A-CHIP mutant are indicated. The U-boxes form the main dimer interface between CHIP protomers and also function as scaffolds for loops known to be involved in protein-protein interactions.

To better understand the allosteric mechanism by which changes in the TPR-domain of CHIP can result in an inhibition of CHIP-mediated ubiquitin discharge from the E2∼Ub complex (Fig 5*D* and 5*E*), we employed high resolution HDX-MS (hydrogen deuterium exchange detected by mass spectrometry) to analyze changes in the solvent accessibility for residues within CHIP as a measure of dynamic conformational differences. Untagged wt and K30A CHIP proteins, purified using conventional chromatography (see supplemental Fig. S2*A*), were incubated in the presence of deuterium for intervals of up to 2 h, quenched and then digested prior to analysis by LC-MS/MS. In all, 67 unique CHIP peptides (Fig 6*A*, supplemental Fig. S5 and Table S1) were identified (98.7% coverage of the protein) and characterized across the time course. The data for the 60s incubation was mapped onto the crystal structure of murine CHIP (Fig 6*B*; 2C2L - there is >97% identity between mouse and human CHIP) and analyzed (32) to give the average deuteration at the single amino acid level (Fig 6*C*). The analysis showed a marked difference in solvent exposure between residues in the TPR-domain of wild-type *versus* mutant CHIP, with the wt TPR displaying much greater incorporation of deuterium than the K30A mutant. The data therefore provide direct evidence that substituting Lys^30^ with Ala stabilizes CHIP structure in a similar manner to that shown previously by Hsp70-based peptides (9). By examining deuteration over time of selected individual peptides spanning the TPR-domain (Fig 6*D*) and U-box (Fig 6*E*) for the wt and the K30A mutant of CHIP, we can see marked differences. For example, there are distinct differences in TPR-domain flexibility, most significantly in helices 2–5 (residues 36–105, salmon pink in Fig 6*F*). Notably, these changes are allosterically communicated to the U-box (red highlight, Fig 6*F*). Thus, mutating Lys^30^ in the TPR-domain to Ala resulted in reduced deuterium incorporation in the U-box domain in addition to the TPR-domain (Fig 6*E*). This result is indicative of a more rigid structure with less flexibility than the wt protein.

The CHIP U-box shows close structural conservation with the RING domains that are present in many E3-ligases (33). Well conserved structural features comprise a β-hairpin connected to a short helix and two capping loops. Multiple X-ray crystallographic representations of this motif have shown that the U-box provides a scaffold for the predominantly hydrophobic interactions between the E3-ligase, the E2-conjugating enzyme and ubiquitin. Our HDX-MS data showed significantly reduced flexibility in both the β-hairpin and the short helix of the U-box motif (residues 239–259) and at the E3-protomer dimer interface (residues 278–293). The recent X-ray structure of the RING protein RNF4, UbcH5a and ubiquitin, has ubiquitin bound to the active site of the E2 with contact made with both protomers of the dimeric RING domain of RNF4 (34). If the equivalent full-length CHIP protein complex exhibits the same binding mode in solution (Fig 6*F*), one would predict that changes to the flexibility of the U-box would have important implications for the transfer of ubiquitin (Ub) from the CHIP-E2-Ub complex to the substrate.

## DISCUSSION

TPR-domains are protein interaction modules present across diverse kingdoms spanning bacteria to mammals, which are studied as scaffolds for the assembly of multi-protein complexes. We demonstrate that the presence of a TPR-domain can pave the way for allosteric regulation through modulation of conformational dynamics. Thus, in keeping with recent conceptual advances on the potential of scaffolds and intrinsic disorder to support allosteric control of signaling complexes (35–37), we show that protein interactions that affect TPR flexibility impact on CHIP structure and regulate its E3-ligase activity.

Recent crystallographic analyses of the Rap proteins from Gram-negative bacteria question the widely held view that TPR-domains have an invariant structure on ligand binding by showing that interaction of the RapJ TPR with PhrC generates large changes in the conformation of the protein as a whole (6). In agreement with a previous study (9) analyzing the TPR-domain in full-length CHIP using HDX-MS (Fig. 6), we find that it is “loosely folded” and that the first 70 amino acids are 100% deuterated within the first 10 s of exposure to D_2_O, indicative of intrinsic disorder (ID). Fluctuation measurements for individual residues in the TPR-domain of CHIP using MD simulations (Fig. 4 and 5) agreed with the HDX-MS, indicating a high degree of flexibility which is significantly reduced upon ligand binding or the introduction of structure stabilizing amino acids. In addition, we see extensive correlations in motions between groups of residues and protein domains. Correlated motions (motion occurring in the same phase) between one TPR and the U-box domains of the dimer, and anticorrelated motion (motion occurring in opposite phases) between the two U-box domains (Fig 5*A*) take place. HDX-MS analysis of wild-type *versus* K30A CHIP reveals that correlated and anticorrelated motions are linked to allosteric regulation of the CHIP U-box. It is striking that ligand binding (Fig. 5; (9)) or substitution of Lys^30^ (Fig. 5 and Fig. 6) suppresses motions within the TPR itself as well as in the U-boxes. Thus, loss of coordinated motion and intrinsic flexibility appear to be key components of the allosteric mechanism by which TPR-binding ligands such as Hsp70 can modulate its activity. The HDX-MS data, together with biochemical assays demonstrating that the K30A mutant retains some catalytic activity, suggest the hypothesis that the K30A mutant of CHIP acts as a functional scaffold but does not have the required conformational plasticity to complete the catalytic cycle competently. This is supported by data showing that K30A-CHIP is still able to bind both UbcH5 and IRF-1, albeit with a reduced affinity (Fig. 5). This could be rationalized by the K30A TPR mutant allosterically locking the U-box motif and stabilizing an intermediate. The K30A mutation clearly illustrates that catalysis can be regulated by the TPR-domain and that chaperone occupancy of the TPR-domain might trigger progression through the catalytic cycle in the wild-type enzyme.

Dynamic protein motion and flexibility are emerging as potential hallmarks of E3-ligase mediated ubiquitination. Studies on cullin-RING E3-ligases have shown that flexibility in substrate-binding proteins and Rbx subunits is required for efficient polyubiquitination. Moreover, the cullins have recently been described as conformationally labile. Together, the flexible components of the cullin-RING E3-ligase complexes function to facilitate a shortening of the distance between the E2 and the substrate to initiate ubiquitination as well as an increase in the E2-substrate distance to accommodate polyubiquitination (38). In another model, flexible regions of the yeast E3-ligase San1 (39) and the ribosome-associated ligase Ltn1 (40) aid in substrate selection by facilitating the recognition of misfolded or defective nascent-polypeptides. Here, we describe a third route by which E3-ligase structural flexibility can regulate ubiquitination. In this case, changes in the degree of TPR-domain secondary structure, flexibility and motion are transmitted to the U-box of CHIP. E2:E3 interactions are critical to the generation of allosteric changes in the E2, which activate the thiolester-linked ubiquitin (34, 38, 41). CHIP in which the TPR has been stabilized is deficient in its ability to bind to both substrate and the E2, resulting in reduced E2∼Ub thiolester discharge and transfer of ubiquitin to the substrate (Fig. 5). Thus, the TPR-domain in CHIP provides the plasticity it requires to act as an E3-ligase but can also act as an “allosteric switch” where the introduction of a more ordered stable structure can “turn off” its E3-function. This could be dependent on the nature of the substrate and/or the degree of “foldedness,” as we have seen that Hsp70 can stimulate the ubiquitination of BAG-1s under the same experimental conditions where it inhibits the modification of p53 and IRF-1. Alternately, it could be that Hsp70 can interact with CHIP in distinct modes and that this depends on the cellular environment or on post-translational factors (25, 42). Our study supports the hypothesis that site-to-site allosteric coupling is enhanced when ID domains are present and when a fold-on-binding mechanism is employed (43, 44). It also demonstrates that intrinsic disorder, scaffolding and allostery can all be linked in a single polypeptide chain as well as in multi-protein complexes.

The current study provides compelling evidence that Hsp70 is not simply acting as a targeting moiety for CHIP in the canonical protein quality control/chaperoning pathways, but is intimately linked to the control of CHIP activity. We demonstrate that Hsp70 can modulate CHIP noncanonical function as a docking-dependent E3-ligase (Fig. 1) by acting as a negative regulator of IRF-1 and p53 ubiquitination. On the other hand, we know that Hsp70 can also stimulate CHIP-mediated substrate modification as it does for BAG-1 (22). However Hsp70 stimulated modification of BAG-1 by CHIP can still be overcome by the Hsp70 C-terminal peptide (Fig 1H), suggesting that Hsp70 could facilitate BAG-1 modification through an interaction with the substrate rather than through binding to the TPR-domain of CHIP. Support for the negative regulation of CHIP by Hsp70 comes from studies on Smad1/5 (45) where Hsp70 inhibits CHIP-mediated ubiquitination and from α-synuclein where suppression of mono-ubiquitination by BAG-5 is Hsp70-mediated (46). Broadening the function of TPR-domains to include allosteric regulatory roles offers the opportunity to modulate the activity of rate-limiting steps in protein homeostasis pathways that are key to healthy aging and which play a significant role in preventing the development of neurodegenerative diseases and cancer. The ability of TPR-domains to accommodate ligands with diverse primary and secondary structures (3, 47, 48) should encourage us to think that TPR-directed biologics and/or small molecules can be identified for specific proteins, offering the potential for allosteric drug development.

## Footnotes

^1^ The abbreviations used are:

TPR: tetratricopeptide repeat
CHIP: Carboxy-terminus of Hsc70-interacting protein.
